# Maternal enrichment increases infantile spatial amnesia mediated by postnatal neurogenesis modulation

**DOI:** 10.3389/fnbeh.2022.971359

**Published:** 2022-08-25

**Authors:** Grecia López-Oropeza, Pilar Durán, Alonso Martínez-Canabal

**Affiliations:** Departamento de Biología Celular, Facultad de Ciencias, National Autonomous University of México (UNAM), Mexico city, Mexico

**Keywords:** postnatal neurogenesis, infantile amnesia, spatial memory, intergenerational regulation, epigenetic inheritance, cognitive development

## Abstract

Infantile amnesia, the inability to form long-lasting episodic memories, is a phenomenon extensively known but with no clear understanding of its origins. However, a recent study showed that high rates of hippocampal postnatal neurogenesis degrade episodic-like memories in infants a few days after memory acquisition. Additionally, new studies indicate that exposure to an enriched environment in mice leads to high hippocampal neurogenesis in their offspring. Nevertheless, it is still unclear how this intergenerational trait affects the persistence of hippocampal memories. Therefore, we evaluated spatial memory retention in the offspring of enriched female mice after weaning to address this question. Ten days after spatial learning, we tested memory retention, observing that the offspring of enriched dams increased spatial memory failure; this finding correlates with high proliferation rates in the hippocampus. Furthermore, we evaluated the causal relationship between postnatal hippocampal neurogenesis and memory failure using the antiproliferative drug Temozolomide (TMZ), which rescued spatial memory retrieval. Finally, we evaluated neuronal activity in the hippocampus quantifying the cells expressing the immediate early gene c-Fos. This evaluation showed engram modifications between groups. This neural activity pattern indicates that the high neurogenesis rates can modify memory engrams and cognitive performance. In conclusion, the inherited increase of hippocampal neurogenesis by enriched dams leads to plastic changes that exacerbate infantile amnesia in a spatial task.

## Introduction

Infantile amnesia is the retention failure of episodic-like memory during the early life stages. This phenomenon depends on the memory fast-forgetting present during postnatal and early infancy stages (Wetzler and Sweeney, 1986). Psychological explanations for infantile amnesia rely on the sense of self (Howe and Courage, 1993), theory of mind (Perner and Ruffman, 1995), and language development (Simcock and Hayne, 2002). However, infantile amnesia appears also in altricial animals (Campbell et al., 1974; Akers et al., 2014), strongly suggesting a biological explanation besides the psychological interpretation. In addition, a predominant idea of such a biological basis is the concept that continuous brain development after birth would impede proper and permanent memory consolidation. A notorious component of postnatal brain development is the high rate of addition of newborn neurons in the hippocampal dentate gyrus. This constant adding of newborn cells would explain the degradation of previously established memory circuits by a continuous rearrangement of hippocampal memory circuits (Josselyn and Frankland, 2012). Previously published works show this hypothesis as correct, probing the impact of postnatal neurogenesis modifications in contextual fear-associated and spatial memory retention and retrieval (Akers et al., 2014; Guskjolen et al., 2017, 2018).

Different mechanisms modulate postnatal and adult neurogenesis, including enhancement by aerobic exercise, environmental enrichment, or antidepressants, while irradiation, stress, and chemotherapy ablate it. However, recent data suggests that it could be an intergenerational mechanism that modulates hippocampal neurogenesis. In correspondence, a study suggests that the offspring of progenitors exposed to aerobic exercise increase their levels of neurogenesis compared to the offspring of non-enriched progenitors (McGreevy et al., 2019). The epigenetic mechanism that regulates intergenerational transmission is still unclear. However, data suggests that the transference of regulatory microRNAs and DNA methylations could contribute to neurogenesis levels modulation (Short et al., 2017). In addition, McGreevy et al. (2019) showed that the offspring of exercised progenitors express behavioral improvements when they reach adulthood; another study showed an improvement in LTP formation in the progeny of progenitors exposed to aerobic exercise (Benito et al., 2018). Nonetheless, no available data shows the impact of intergenerational neurogenesis modulation on infantile amnesia expression.

We previously hypothesized (Martinez-Canabal et al., 2019) that the offspring increase in neurogenesis derived from aerobic exercise and environmental enrichment of progenitors can exacerbate infantile amnesia. In the present study, we show that environmental enrichment of dams before pregnancy leads to offspring with higher rates of spatial memory forgetting; hippocampal neurogenesis is causally responsible for this effect which also involves a comprehensive modification of hippocampal cells recruitment in memory retrieval.

## Material and Methods

### Animals and housing

C57BL6/J females were obtained from the Science Faculty breeding colony and were separated into two groups. The first batch of dams was grouped in an enriched environment house-caged of 30 × 32 × 20 cm dimensions with a running wheel for two months. After that, the second batch of dams was housed in standard acrylic cages of 32 × 16 × 14 cm simultaneously. All dams had free access to water and food (pellets of 5008 or 5001, LabDiet^®^). After that, all dams were caged with males in standard acrylic cages for 15 days and then housed alone for breeding. Two males were used in each experiment and counterbalanced between experimental groups when possible.

Offspring of dams were weaned at 21 days postnatal age and housed in standard conditions. All animals were maintained in rooms with controlled temperature (22 ± 2°C) and humidity (40%–60%) in a 12:12 light/dark cycle during all experimental time. The local ethics committee approved all the experiments carried out in this project (CEARC/Bioética/02032021).

### Water maze apparatus

The offspring of all dams were trained in the spatial version of the Morris water maze, where they had to find a dry acrylic platform of 10 cm in diameter in a swimming pool of 120 diameter × 40 cm in height. The swimming pool’s water was dyed with white non-toxic acrylic paint to block the platform’s visibility; the water’s temperature was maintained at 24 ± 1°C. Four visual cues were located on the room’s walls to orient the mice to find the platform. Each cue is 90 cm wide square printed in B/W with very discreet symbols: “X”, “**|||**”, “●”, “≡”; from the center of the maze, the cues occupy a 40° angle which represents 44% of the visual space around the poll. This layout aims to facilitate the visual detection of the cues, considering that the visual system acuity in weanling mice is still in development between P21 and P30. All the mice activity was recorded with a webcam fixed on the ceiling over the water maze center, and all the videos were recorded and analyzed with the software Anymaze^®^5.1.

### Training protocol

Each pup was collocated into the maze randomly from different starting points per trial and let swim to the platform where pups were allowed to stay 15 s; if they did not reach the platform after 60 s, they were gently located over it. Pups were allowed four trials per day from different starting points. After 24 h of the last trial, pups underwent a probe test where the platform was removed to observe whether pups had established a spatial map of the platform location. At the probe test all mice were released from the same point. During the probe test, the percentage of time spent in four zones of 20 cm diameter around the platform and equivalent areas were used to determine the correct search pattern of the mice. Additionally, the x, y coordinates of all the mice from each experimental group were tracked to generate Heatmaps using MatLab R2021b^®^. The releasing point is located in the bottom of the depicted heatmap while the platform site is located within the inner circle. Finally, ten days after the first probe test, pups were given a second probe test to evaluate memory retention. For the Open field test, mice were placed during 10 min in a flat arena of 75 cm per side with walls of 20 cm. Mice could roam freely during the trial, and the distance traveled was measured using the same system as in the water maze.

### Neurogenesis ablation

Temozolomide (TMZ; Sigma-Aldrich^®^, PHR1437) administered subcutaneously (25 mg/kg in saline with 10% DMSO) was used to ablate hippocampal neurogenesis in pups after the first probe test. Injections were administered twice daily on days 1–4 and 7–10 (Niibori et al., 2012; Martinez-Canabal et al., 2013). Garthe et al. (2009) showed that using a similar protocol chronically; there was no decrease in erythrocytes, no increase in microglia, and no impairment in motor function or swim ability. The only detectable side effect is decreased leucocytes after a month of treatment. However, mice health was monitored daily, and it was only used for 10 days in the present work.

### Immunohistochemistry

Ninety minutes after the second probe test, pups were sacrificed using an overdose of sodium pentobarbital (210 mg/kg., Pisabental^®^) and transcardially perfused with 0.9% cold saline and paraformaldehyde (PFA, 4%). Afterward, brains were collected and post-fixed in PFA 4% and then transferred to 30% sucrose solution. Brains were mounted and sliced using Cryostat (Leica CM1860) at -20°C into 50 μm slices and immediately collected at intervals of 300 mm in antifreeze solution (40% glycerol, 10% ethylene glycol in PBS). Then, a heating antigen retrieval protocol was applied to all slices using citric acid for anti-ki67 and anti-DCX antibodies (10 mM Citric Acid, 0.05 Tween 20, pH 6.0) or EDTA for anti-c-Fos antibody (1 mM EDTA, 0.05% Tween, pH 8). Next, brain sections were removed from slides and located in culture plates with blocking solution (Bovine Serum Albumin, 2%, Santa Cruz Biotechnology, NGS 2%, Jackson Immunoresearch in TBS-T) for 60 min. Subsequently, slices were incubated for 24 h with primary antibodies (1:4,000 Monoclonal Rabbit anti-DCX #4604S, Cell Signaling Technology^®^; 1:4,000 Monoclonal Rabbit anti-ki67 Abcam^®^ AB15580; or 1:2,000 Polyclonal Rabbit anti-c-Fos, Abcam EPR21930-238) at 25°C. On a second day, slices were incubated in a secondary antibody (1:2,000 111-065-003 Biotin-SP-conjugated AffiniPure Goat Anti-rabbit) for 60 min and then incubated in avidin-biotin complex (1:250, kit ABC-peroxidase, elite VECTASAN^®^, Vector) for 60 min. Finally, tissue was revealed using DAB-Ni (0.05%; Sigma) with 025% H_2_O_2_ (Sigma), and slices were mounted and counterstained with nuclear staining methyl green for further analysis.

### Cell counting and analysis

We manually quantified, employing Tally Counter, the number of DCX+ cells and ki67+ cells using a Primo Star3 microscope (Zeiss) using the 100× objective (N.A. 1.25). Then, photographs of D.G. of each section were taken with a 10× (N.A. 0.25) objective to measure the area and calculate the density using the image analysis software ImageJ^®^ (1.48 v). On the other hand, c-Fos positive nuclei were measured by collecting photographs of D.G., CA3, CA1, and SUB ventral and dorsal regions with 10× objective (N.A. 0.25) using ImageJ for automatic cell counting, the analysis parameter to include c-Fos positive nuclei were: 6–60 μm area and 0.7–1 circularity.

### Statistical analysis

All animal groups were analyzed using, when appropriate, unpaired Student’s two-tailed *t*-tests, repeated-measures analysis of variance with Tukey *post hoc* comparisons if necessary, or Pearson correlation coefficient in Statistica64^©^ (12 v).

## Results

### The offspring of enriched dams show exacerbated infantile amnesia in a spatial memory task

**Figure 1:**
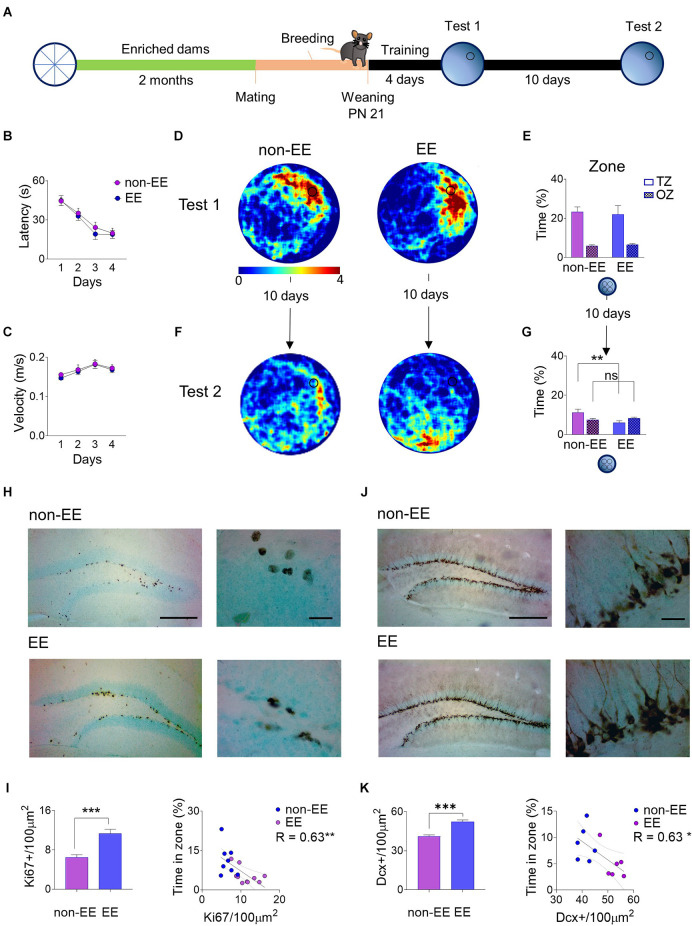
Female mice were exposed to an enriched environment for 2 months; after that, they were mated with males and allowed to breed. P21 pups were trained in the water maze, given one probe test and a second retention probe test after 10 days. **(A)** Control mice of the same age (non-EE) and the offspring of enriched dams (EE) learned at the same rates during training **(B)** and showed the same swimming speed **(C)**. At the first probe test, non-EE and EE mice swam the same times around the former platform location, showing both heatmaps (the stronger color depicts up to four crosses in the area) **(D)** and the time spent in the correct zone vs. other zones **(E)**. However, 10 days later, there was a reduction in the time spent around the original platform site in both groups. Nevertheless, the heatmaps showed a notoriously higher reduction in the EE group **(F)**. The time spent in the correct zone vs. other zones decreases more in the EE group than in the control group **(G)**. The proliferation marker Ki67 appears in the subgranular zone of both groups but notoriously more in the EE group showing the characteristic nuclear expression arranged in cell clusters **(H)**. The density of Ki67+ cells is higher in the EE group showing a negative correlation between density and the performance in the second probe test **(I)**. The immature neuronal marker Doublecortin (DCX) also appears in high numbers in the subgranular and granular zones of the dentate gyrus, more notorious in the EE group **(J)**. The cell density of DCX+ somata is higher in the EE group simultaneously, and it also shows a negative correlation with the time spent in the correct zone on the probe test **(K)**. **P < 0.01 Tukey *post-hoc*; ***P < 0.001 student’s t, *P < 0.05, **P < 0.01 slope deviation from zero, scale bar 250 μm low magnification, 20 μm high magnification. Behavior (non-EE *n* = 11; EE *n* = 11), Ki67 analysis (non-EE *n* = 8; EE = 10). DCX analysis (non-EE *n* = 6; EE *n* = 6).

At 21 days after birth, pups from enriched dams (EE) and controls (non-EE) were trained to locate a hidden platform in the water maze with a 4-day training (Figure 1A). This age was chosen based on previous works showing that between P20 and P24, mice can be trained in the water maze paradigm and still show infantile amnesia few days later (Barnhart et al., 2015; Guskjolen et al., 2017). During training there was a noticeable decrease in the latency to reach the platform across days (*F*_3,60_ = 28.00, *P* < 0.001) but not a difference between groups (*F*_1,20_ = 0.35, *P* = 0.56; Figure 1B), both groups swam at the same speed along the training days (*F*_1,20_ = 0.12, *P* = 0.72; Figure 1C). Twenty-four hours after the last training day, a probe test was given, allowing pups to swim in the pool without a platform. Tested pups showed a preference for the platform’s zone, as shown with path-crossing density (Figure 1D). In addition, mice showed a strong preference for the 20 cm radius zone (*F*_1,20_ = 32.44, *P* < 0.001) where the platform was located compared to the other three equivalent zones in the maze. However, there was no difference in the preference for the target zone between groups (*F*_1,20_ = 0.03, *P* = 0.85; Figure 1E). The ability to form long-term spatial memories was not affected by the litter; a comparison of the spatial performance of each litter showed no difference (*F*_5,16_ = 0.04, *P* = 0.99); also, the comparison between litters showed variance homogeneity (Levene’s test: *F*_5,16_ = 1.48, *P* = 0.24). The mice were again tested ten days after the first probe test, and both groups showed a decrease in spatial memory predicted by infantile amnesia and observed in heatmaps by a more dispersed search pattern. In the case of the EE the higher density appears in sites near the releasing point and not around the target area (Figure 1F). Furthermore, there was a decrease in the correct zone searching by the EE mice in comparison with the non-EE controls (Group × Zone: *F*_1,20_ = 5.37, *P* = 0.03, *post-hoc* non-EE vs. EE: *P* = 0.009; Figure 1G).

Proliferation rates were evaluated by quantifying cells immunoreactive to Ki67, a universal proliferation marker that labels all active cell cycle phases (Kee et al., 2002). The cells appear in well-defined clusters along the subgranular zone in both groups, with a noticeable higher immunoreactivity in the EE group (Figure 1H). The cell counting corroborated a higher number of Ki67+ cells in the EE group (*t*_16_ = 4.41, *P* < 0.001) by 74.42% (Figure 1I). Additionally, the number of Ki67+ cells measured in each animal showed a negative correlation with the time spent by the mice in the correct zone of the water maze probe test performed ten days after training (*R* = -0.63, *P* < 0.01), suggesting that the more newborn cells, the strongest the spatial forgetting (Figure 1I).

The density of immature neurons in the dentate gyrus was evaluated by quantifying immunoreactivity to Doublecortin (DCX), a microtubule-associated protein, a universal marker of immature neurons (Francis et al., 1999). The immature neurons appear in the granule cell layer closer to the subgranular zone in large amounts, consistent with high rates of neuron generation in early life stages, showing conspicuous dendritic arbors and axons towards CA3 (Figure 1J). The cell counting corroborated a higher number of DCX+ cells in the EE group (*t*_10_ = 6.36, *P* < 0.001) by 27.22%. Likewise, the number of DCX+ cells measured in each animal showed a negative correlation with the time spent by the mice in the correct zone of the water maze ten days after training (*R* = -0.63, *P* < 0.03), suggesting that the more immature neurons, the strongest the spatial forgetting (Figure 1K). The increment in basal neurogenesis levels in the progeny of enriched dams is not related to higher levels of spontaneous activity. Spontaneous locomotor activity was measured in the open field and during a 10-min trial, both the offspring of enriched dams and control animals traveled the same distance (non-EE: 55.98 m ± SEM 4.27, EE: 55.66 ± SEM 1.93, *t*_17_ = 0.05, *P* = 0.95).

### Ablation of postnatal neurogenesis in the offspring of enriched dams rescues infantile spatial amnesia

**Figure 2:**
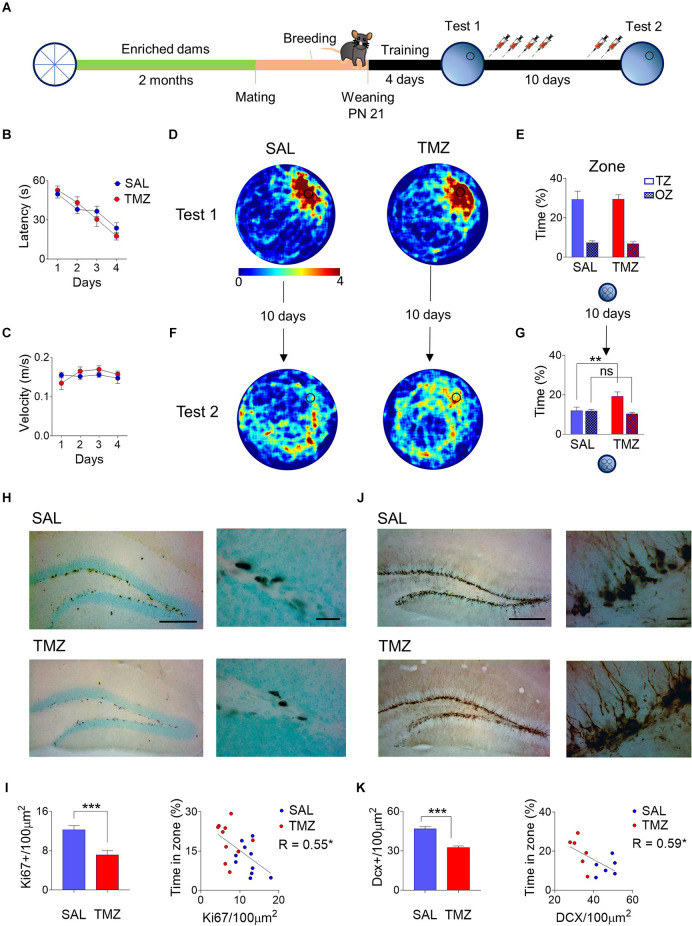
Female mice were exposed to an enriched environment for two months; after that were mated with males and allowed to breed. P21 pups were trained in the water maze, given one probe test, and treated with temozolomide (TMZ) with a second retention probe test after 10 days. **(A)** Control mice vehicle-injected of the same age (SAL) and the offspring of enriched dams (EE) learned at the same rates during training **(B)** and showed the same swimming speed **(C)**. At the first probe test, SAL and TMZ mice swam similar paths across the former platform location, showing both heatmaps **(D)** and the time spent in the correct zone vs. other zones **(E)**. However, 10 days later, there was a reduction in the time spent around the original platform site in both groups. Nevertheless, the heatmaps showed a notoriously higher time around the platform location in the TMZ group **(F)**. The time spent in the correct zone vs. other zones was higher in the TMZ group than in the SAL group **(G)**. The proliferation marker Ki67 appears in the subgranular zone of both groups but notoriously more in the SAL group, showing the characteristic nuclear expression arranged in cell clusters **(H)**. The density of Ki67+ cells was lower in the TMZ group showing a negative correlation between density and the performance in the second probe test **(I)**. The immature neuronal marker Doublecortin (DCX) also appears in high numbers in both groups’ subgranular and granular zones of the dentate gyrus **(J)**. The cell density of DCX+ somata was lower in the TMZ group simultaneously, and it also shows a negative correlation with the time spent in the correct zone on the probe test **(K)**. **P < 0.01 Tukey *post-hoc*; ***P < 0.001 student’s t, *P < 0.05, **P < 0.01 slope deviation from zero. Scale bar = 250 μm low magnification; 20 μm high magnification. Behavior (SAL *n* = 10; TMZ *n* = 10), Ki67 analysis (SAL *n* = 10; TMZ = 10). DCX analysis (SAL *n* = 6; TMZ *n* = 6).

The offspring of dams exposed to environmental enrichment for two months showed an accelerated forgetting of spatial memory. To determine if the fast decline in memory is causally associated with the observed high rates of neurogenesis, we ablated neurogenesis with TMZ in a group of pups born from previously enriched dams. In a control group of pups, offspring of enriched dams received only vehicle (SAL) (Figure 2A). Each group contains mice from all the litters used for the experiment. At Twenty-one days of age, pups were trained to locate a hidden platform in the water maze, showing consistent learning to reach the platform across training days (*F*_3,54_ = 28.46, *P* < 0.001) with no difference between groups in times to reach the platform (*F*_1,18_ = 0.08, *P* = 0.78; Figure 2B) or swimming speed (*F*_1,18_ = 0.23, *P* = 0.63; Figure 2C). After training, the animals performed a probe test without a platform in which both groups showed constant crossings over the area where the platform was previously located, as depicted in the heatmaps (Figure 2D). Both groups showed robust spatial retrieval with strong preference for the target zone (*F*_1,18_ = 72.34, *P* < 0.001) with no differences between SAL and TMZ (*F*_1,18_ = 0.01, *P* = 0.9; Figure 2E). Animals were tested for spatial memory retention ten days after training; in this probe test, there was a memory rescue in the TMZ group compared to the SAL group (Group × Zone: F_1, 18_ = 8.08, *P* = 0.01; target zone *post hoc* comparison Sal vs. TMZ *P* = 0.008), the TMZ group also showed searching discrimination between the target zone and other zones (*post hoc* TZ vs. OZ: *P* = 0.004; Figures 2F,G).

The mice in the TMZ group showed a decrease in proliferation rates compared with the SAL. This was expected because TMZ is a drug designed to target neural progenitor cells in S-phase (Ortiz et al., 2021). As expected, proliferating cells Ki-67+ showed normal morphology organized in clusters and located over the subgranular zone; however, the decrease in numbers was qualitatively noticeable compared to the SAL group (Figure 2H). The density quantification of Ki-67+ cells showed a decrease of 41.61% in the SGZ (*t*_18_ = 4.35, *P* < 0.001). Interestingly, linear regression analysis showed a negative correlation between the density of proliferating cells and the water maze performance (*R* = -0.55, *P* = 0.01; Figure 2I). The immature neurons DCX+ appear copiously in the granule cell layer close to the subgranular zone, consistent with the large neuron generation in early life stages, showing conspicuous dendritic arbors and mossy fibers. Nevertheless, fewer immature neurons appear in the TMZ group (Figure 2J). The quantification corroborated a lower density of DCX+ cells in TMZ mice (*t*_10_ = 6.385, *P* < 0.001) by 30.47%. The number of DCX+ cells showed a negative correlation with the time spent by the mice in the correct zone of the water maze (*R* = -0.63, *P* = 0.03). This correlation suggests that a decrease in the number of immature neurons contributes to the spatial memory rescue (Figure 2K).

### Neurogenesis ablation in mice descendant from enriched dams decreases active hippocampus cells during spatial retrieval

**Figure 3:**
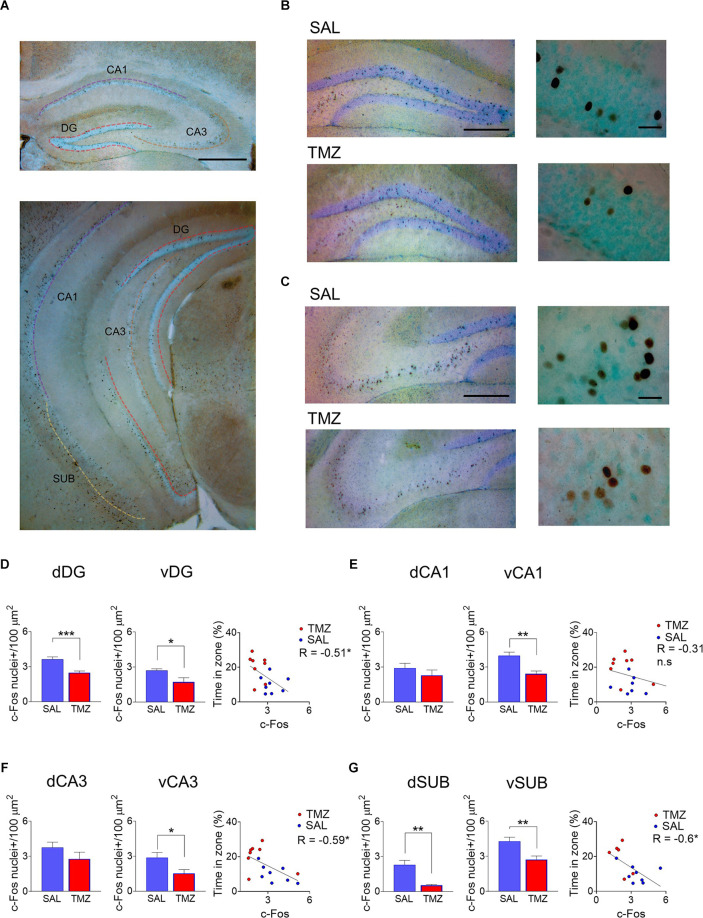
The activity levels in the hippocampus at the second probe test were evaluated by quantifying immediate early gene expression c-Fos positive nuclei. The hippocampus was subdivided in dorsal and ventral divisions and in its different layers dCA1, vCA1, dCA3, vCA3, dDG, vDG, dorsal subiculum (dSUB), ventral subiculum (vSUB). **(A)** Here it is shown a representative picture of low and high magnification of both Dentate gyrus granule cell layer **(B)** as CA3 pyramidal layer **(C)** in both areas is notorious a conspicuous c-Fos expression in the whole area and exclusively in the nuclear and perinuclear zone of the cells. Qualitatively more cells are observed in the SAL pictures vs. the TMZ groups. In both the hippocampus’s dorsal and ventral divisions, the SAL animals have a higher density of c-Fos+ cells. Linear regression analysis showed a negative correlation between the density of c-Fos+ cells and the time the mice spent in the target zone in the water maze at the second probe test **(D)**. In CA1, there was no difference between SAL and TMZ in c-Fos+ cells density; also, no correlation was observed **(E)**. In both the dorsal and ventral hippocampus, the density of CA3 c-Fos+ cells is higher than in the TMZ groups; a linear regression showed a negative correlation between the c-Fos+ cells and the spatial performance in the water maze **(F)**. In both dorsal and ventral subiculum, c-Fos+ cells density is higher in SAL than in the TMZ group; a linear regression showed a negative correlation between the c-Fos+ cells and the spatial performance in the water maze **(G)**. *P < 0.05, **P < 0.01, ***P < 0.001 student’s t. Scale bar = 500 μm panoramic magnification; 250 μm low magnification; 20 μm high magnification. DG (SAL *n* = 9; TMZ *n* = 8), CA1 (SAL *n* = 9; TMZ = 8), CA3 (SAL *n* = 9; TMZ *n* = 7), Subiculum (SAL *n* = 8; TMZ *n* = 5).

After the second probe test, the mice with ablated neurogenesis (TMZ) and their vehicle control group (SAL) were analyzed for expression of the immediate early gene protein c-Fos. Immunoreactive c-Fos nuclei were observed in the granule layer of the dentate gyrus and the different pyramidal subdivisions of the hippocampal cornu ammonis in dorsal and ventral regions (Figure 3A). The c-Fos staining appears strongly specific inside and around the cell nucleus sparsely in the whole hippocampus. Consequently, the counterstaining allowed us to remark the limits of every area. Despite the high number of stained nuclei in every area and animal studied, there is a trend toward more density of immunoreactive cells in the animals not treated with TMZ (Figure 3B).

The c-Fos+ nuclei quantified in the dentate gyrus showed a density decrease in the treated animals both in dorsal (*t*_15_ = 4.453, *P* < 0.001) and ventral (*t*_14_ = 2.75, *P* = 0.02) areas. Moreover, the density of active neurons in the dentate gyrus negatively correlates with the spatial memory retrieval in the second probe test (*R* = -0.51, *P* = 0.04; Figures 3B–D). Correspondingly, the density of c-Fos+ nuclei in the CA3 layer showed an increase in the neurogenesis ablated animals, both in dorsal (*t*_15_ = 4.03, *P* = 0.001) and ventral (*t*_13_ = 2.47, *P* = 0.03) areas. The density of CA3 active cells correlated negatively with the spatial memory retrieval test (*R* = -0.59, *P* = 0.02; Figures 3C–F). Contrariwise to DG and CA3, CA1 layer did not show a difference in active cells between neurogenesis ablated animals and vehicle treated in dorsal (*t*_15_ = 0.98, *P* = 0.33) and ventral (*t*_14_ = 1.37, *P* = 0.19) areas. Whole CA1 active cells density did not show a significant correlation with spatial retrieval (*R* = -0.31, *P* = 0.23; Figure 3E). Notably, the dorsal subiculum showed an increase of active cells in the animals with ablated neurogenesis (*t_11_* = 3.59, *P* = 0.004) the same as the ventral subiculum (*t_11_* = 3.11, *P* = 0.009). Finally, whole subiculum active cells correlated negatively with spatial memory retrieval (*R* = -0.6, *P* = 0.02; Figure 3G).

## Discussion

The present study confirmed that the offspring of dams exposed to an enriched environment produce more newborn neurons during postnatal stages than the offspring of non-enriched dams. In agreement with the neurogenic hypothesis of infantile amnesia, these mice showed lower spatial memory retention ten days after training before reaching adulthood. Furthermore, we showed that neurogenesis ablation induces a partial rescue of the spatial memory, corroborating the causal relationship between decay in spatial retention and neurogenesis. In parallel, we observed an increase in the number of active cells in the hippocampus of animals with inherited high neurogenesis compared to those mice with ablated neurogenesis. This finding supports the idea that high neurogenesis rates in postnatal animals modify the integrity of the memory supporting circuits and lead to spatial memory failure after initial memory acquisition.

Environmental enrichment with access to aerobic exercise improves the generation of new hippocampal neurons (van Praag et al., 1999a, b); however, the idea that the offspring inherits this trait was only recently proposed (McGreevy et al., 2019). This discovery prompts the need to understand the behavioral implications of the transgenerational increase of newborn neurons. Accordingly to extensive literature, the hippocampal postnatal and adult newborn neurons have a role in distinct cognitive tasks, like pattern separation (Aimone et al., 2011), episodic-like memory forgetting (Feng et al., 2001; Akers et al., 2014), cognitive flexibility (Epp et al., 2016), and resilience to depression and stress (Snyder et al., 2011). Therefore, we have focused the present study on the implication of the inherited neurogenesis increase over memory forgetting appearing in postnatal stages, commonly known as infantile amnesia.

We observed a faster degradation of spatial memory in the offspring of enriched dams. At the same time, we observed an increase in both cell proliferation in the postnatal dentate gyrus and the number of newborn immature neurons, a discovery consistent with the idea that the continuous brain development during infancy, in this case by the addition of new functional hippocampal neurons, can lead to memory failure during infancy (Josselyn and Frankland, 2012). Additionally, the observed negative correlations between measured neurogenesis and the spatial memory retrieval indicate a possible implication of the inherited increase in neurogenesis to the enhanced spatial memory retrieval failure. Using Temozolomide, a chemotherapy drug capable of ablating neurogenesis without adverse reactions, we established a causal relationship between inherited neurogenesis and the increase of infantile spatial amnesia. According to previous studies, factors such as the DNA methylation in critical genes or the transmission of regulatory microRNAs can drive the intergenerational neurogenesis modification (McGreevy et al., 2019). However, if those epigenetic factors are responsible for the neurogenesis increase in the offspring of enriched progenitors, in the present study, we show for the first time the importance of epigenetic inheritance on infantile amnesia driven by hippocampal neurogenesis.

Previous works highlighted the importance of distinguishing between engram degradation and a recall failure in infantile amnesia (Travaglia et al., 2016; Guskjolen et al., 2018). The present study corroborates the presence of an engram failure in infantile amnesia due to an enlarged number of active cells in critical hippocampal layers. Surprisingly, the number of active cells quantified correlates negatively with the observed memory performance. This finding suggests that the substantial number of newborn neurons in the immature hippocampus generates an excessive noise in the circuit, preventing an optimal engram recall. General disorganization and degradation of the original engram or hiding of the proper engram behind an excessive noise caused by the highly active newborn neurons and the constant addition of new synaptic contacts in the perforant and mossy fiber could lead to the engram recall failure (Meltzer et al., 2005; Toni and Schinder, 2015). In the present study, we showed that decreased and likely more tunned neural activity in the infant hippocampus could promote better memory recall.

In recent years several studies have explored the biological basis of infantile amnesia; e.g., Akers et al. (2014) showed a rescue of infantile contextual memory when postnatal neurogenesis ablated. Furthermore, Guskjolen et al. (2017) showed that infantile amnesia in mice also affects spatial memory retention. The present study confirms that increased neurogenesis in pups promotes forgetting of spatial memory and that neurogenesis ablation can rescue the spatial memory. In this scope, Guskjolen et al. (2018) aimed to recover infant memories using an optogenetic recall method; the study suggests that infantile amnesia is partially due to both a recall deficit and memory erasure. The present study confirms substantial modifications of the hippocampal memory engram by quantifying active neurons; however, there is not enough information to determine the extent the memory storage degradation or a recall failure causes this phenomenon.

The present study adds valuable information to the current knowledge of inherited epigenetic traits to cognitive development. A recent study shows that the offspring of enriched progenitors have an enhanced LTP formation in the adult hippocampus, dependent on microRNAs inherited through gametes (Benito et al., 2018). The same as cognitive tasks enhanced in adult mice with enriched progenitors (McGreevy et al., 2019). These works, together, show that the epigenetic inheritance from progenitors exposed to environmental enrichment implies an enhancement of synaptic plasticity in the offspring evaluated in different paradigms. Furthermore, Guskjolen et al. (2017) showed that infant mice present stronger reversal memory than adult mice, which can lead to the interpretation that cognitive flexibility is a by-product of infantile amnesia, adding infantile amnesia modulation to the list of synaptic plasticity enhancement effects derived from epigenetic inheritance.

In the present study, we evaluated the cognitive effects of the inherited increase of neurogenesis in the offspring. The cognitive implications of this finding are vast; i.e., in the correct interpretation of cognitive differences between populations of the same species based solely on their intergenerational history, both in animal research models, domestic animals, or even humans. Furthermore, the present study implies that some inherited traits might strongly influence infantile learning and memory structure. This hypothesis can help parents and educators to evaluate better educational strategies based on the intergenerational history of preschool and elementary school students.

Nevertheless, the importance of cognitive plasticity led by the generation of newborn neurons is not limited to memory modulation; e.g., newborn neurons added to hippocampal circuits modulate resilience to anxiety or depression. Therefore, the finding that activities and lifestyle of progenitors can eventually lead to resilient offspring to mental health problems is a critical finding that, after further investigation, can derive in establishing health policies to prevent mental health problems even before pregnancy.

Expanding this knowledge should clarify the true extent of the synaptic plasticity modification associated with the increase in neurogenesis by progenitors’ inheritance and how it can affect the lives of animals and even humans. However, despite the possibility of observing various behavioral effects at different life stages, the engram modification and the question of recall failure or memory storage failure remains a complex problem. The data obtained in this study of cell activity shows substantial alterations in the engram structure associated with infantile amnesia; however, we cannot infer from this data if we are observing a failure in the storage of memory, memory recall, or both.

The present study focuses on the role of postnatal newborn neurons on infantile amnesia; however, infantile amnesia is a complex phenomenon that could involve other mechanisms within and outside the hippocampal formation. Travaglia et al. (2016) showed that the switch between NMDA receptor subunits from 2B to 2A could be critical for the change in the capabilities of the hippocampus to store long-term memories. Other molecular changes development-associated can play an unknown role in the memory failure that appears in the first life stages. Outside the hippocampal formation, the prefrontal cortex, which is critical for consolidation (Kitamura et al., 2017), is not entirely developed when infantile amnesia occurs and still lacks complete development of neurons, both excitatory and inhibitory transmission, and the formation of critical connections with distal areas of the brain including the hippocampus (see references in Klune et al., 2021). If the enrichment of progenitors could change the rates at which the prefrontal cortex matures has not been studied yet and could contribute to a broader comprehension of infantile amnesia.

A significant obstacle to studying the roles of areas outside of the hippocampus on infantile amnesia is that this phenomenon only affects episodic-like memories; the only episodic-like memory paradigm hippocampus-independent during consolidation is the conditioned taste aversion. In previous reports, conditioned taste aversion retention after a period of time showed resistance to degradation led by neurogenesis (Akers et al., 2014). Interestingly, in previous works, conditioned taste aversion also showed resistance to degradation caused by infantile amnesia (Campbell and Alberts, 1979; Ueji and Yamamoto, 2012). Likewise, cued fear conditioning acquisition is hippocampal-independent; however, consolidation is not (Oh and Han, 2020). Therefore, understanding the roles of brain circuits other than the hippocampus on infantile amnesia modulation would require different strategies, including optogenetics, electrophysiology, or functional imaging, to study different areas, even using a hippocampal-dependent memory paradigm. In the scope of the present results, it would be relevant to isolate a possible impact of an intergenerational inheritance on infantile amnesia acceleration, modulated by regions other than the hippocampus.

From the data presented in this work, we conclude that the neurogenesis increase in postnatal stages derived from the access to an enriched environment by progenitors leads to an exacerbation of infantile spatial amnesia likely linked to an increase in hippocampal activity.

## Data Availability Statement

The raw data supporting the conclusions of this article will be made available by the authors, without undue reservation.

## Ethics Statement

The animal study was reviewed and approved by Comisión de Ética Académica y Responsabilidad Científica (CEARC). Facultad de Ciencias, Universidad Nacional Autónoma de México (UNAM). This project was made under the approval of the local ethics committee (CEARC) with the number CEARC/Bioética/02032021.

## Author Contributions

A-MC and PD designed the research project. GL-O and AM-C performed experiments and collected data. GL-O and AM-C analyzed data. AM-C, GL-O, and PD discussed data, wrote the article, and prepared Figures. All authors contributed to the article and approved the submitted version.

## Funding

We appreciate the local funding to PD and AM-C, also the DGAPA-PAPIIT projects IA207419 and IA207521 to AM-C.
